# THE NUTS AND BOLTS OF NIH PEER REVIEW: THE CENTER FOR SCIENTIFIC REVIEW

**DOI:** 10.1093/geroni/igz038.763

**Published:** 2019-11-08

**Authors:** Elia Femia, Dana Plude, George W Rebok

**Affiliations:** 1 National Institutes of Health, Bethesda, Massachusetts, United States; 2 National Institute on Aging/National Institutes of Health, Bethesda, Maryland, United States; 3 Johns Hopkins Bloomberg School of Public Health, Baltimore, Maryland, United States

## Abstract

The National Institutes of Health is the largest public funder of biomedical and bio-behavioral research in the United States. The mission is to enhance health, lengthen life, and reduce illness and disability. To achieve this mission, the NIH provides support for cutting-edge research and technology development in a variety of fields, ranging from translation of innovative ideas in technology to basic science on major health challenges and disease. There are many types of research and training opportunities and technology development programs that are supported by the NIH across the 24 institutes and centers that provide funding. The majority of grant applications are reviewed by the NIH Center for Scientific Review (CSR). In this symposium, attendees will get 1) an overview of the types of applications submitted to the NIH for support; 2) the basics of the NIH peer review process and criteria and scoring system for evaluating applications, and 3) tips for writing a more successful grant application. Peer review is the cornerstone of the NIH grants process, and an insider’s view can lead to a better understanding of how the most meritorious projects are identified that lead to innovative re-search in the biomedical and bio-behavioral sciences.

